# Estimating the incidence and mortality of cancer in Colombia: What are the best data for public policies?

**Published:** 2016-06-30

**Authors:** Luis Eduardo Bravo

**Affiliations:** Director of the Cancer Registry of Cali. Department of Pathology, Universidad del Valle, Cali, Colombia; Escuela de Medicina. Facultad de Salud. Universidad del Valle, Cali, Colombia

Colombia simultaneously faces the challenge of controlling both the communicable and non-communicable diseases. Information provided by the population-based cancer registries (PBCRs) of Colombia 1-4 indicates that cancer is a major cause of morbidity in our region. Based on the information provided by the PBCRs of Colombia and taking into account mortality data from cancer, the Colombian National Cancer Institute (NCI) estimates that, in Colombia, there are about 63,000 new cases and 33,000 deaths by cancer each year 5. The number of people living with the disease is unknown. The possibility of reducing cancer mortality and preventing 30% of malignancies has encouraged the Colombian government to design a Cancer National Information System (Cancer-NIS) 6 and to formulate a ten-year plan for its control 7. 

Having good quality statistics on the cancer burden is essential to make an accurate diagnostic of the cancer problem and to design, implement and monitor control measures. Otherwise, the human, social and economic costs produced by this group of diseases will continue to increase, and they will exceed the country's capacity to fight them.

**Figure 1.**
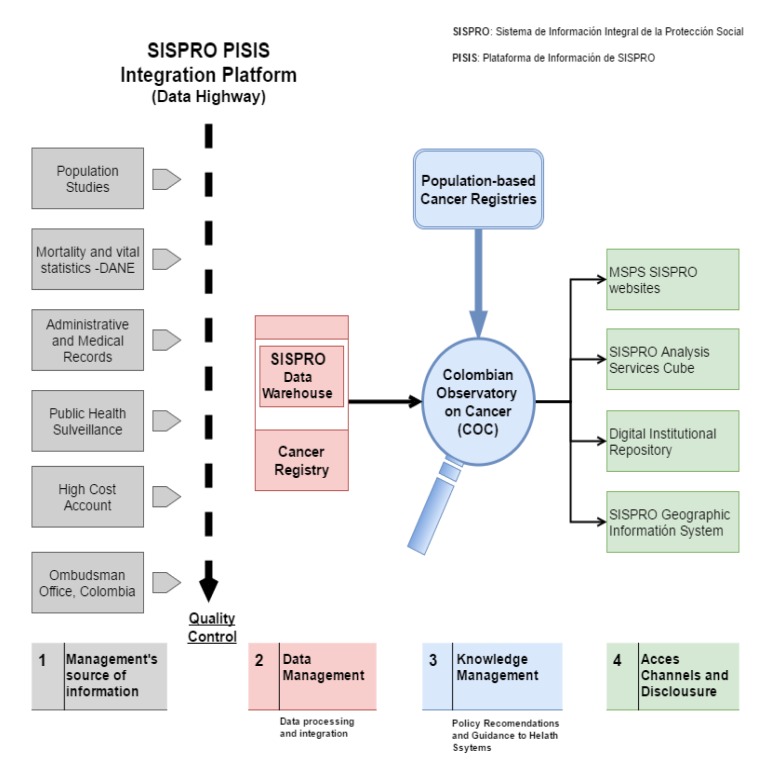
Cancer National Information System of Colombia (Cancer-NIS), (Resolution 4496, 2012). SISPRO: integrated information system of social protection. PISIS: Information Platform of SISPRO

The Cancer-NIS is composed by information sub-systems and inter-sectorial actions that seek to optimize the use of information that are mandatory by legal standards 6 ( Fig. 1). The high cost account (HCA) of the Colombian Ministry of Health and Social Protection (MHHS), one of the Cancer-NIS's sources of information, has released preliminary information on cancer incidence and cancer care in Colombia 8. The HCA reports incidence rates for some of the most common sites of cancer (prostate, breast, colorectal, stomach and lung) that are up to five times lower compared to the ones observed by the Colombian PBCRs 1-4; and the estimates made by the Colombian-NCI 5 and the International Agency for Research on Cancer (IARC) 6. For acute lymphoid leukemias, Cali's figures reported by the population-based cancer registry of Cali are 8 times higher than those reported by the HCA.

There are now four population-based cancer registries (PBCR) in Colombia) that are recognized internationally by the IARC and provide the highest quality incidence data in Bucaramanga, Cali, Manizales y Pasto . These PBCRs collect and classify information on all new cancer cases of permanent residents to produce valid statistics on cancer incidence, patterns, trends and survival 9-11. Colombia is the only country among low- and middle-income countries that has reported accurately the situation of cancer in Cali continuously for 50 years, through the Cancer Registry of Cali with high quality information included in all the volumes of Cancer Incidence in Five Continents 7. The information has been consistent over time and the expected values ​​are comparable with those reported by cancer registries that serve similar populations. This information has allowed the Colombian-NCI to plan control strategies, to set baseline indicators for the current Ten-year Plan for Cancer Control, and to make incidence estimates based on mortality for 90% of the Colombian regions that do not have coverage of cancer registries.

The information disclosed by the HCA shows clear evidence of underestimation of the risk of cancer in the Colombian population. The probable causes are many, and include: inadequate integration of information provided by the various sources of Cancer-NIS, a case definition different from the one established by WHO, lack of precision in the population at risk, insufficient standardization of processes, deficiencies in the training of personnel responsible for reporting of cases, sub-registration of cases, lack of validation by active search of cases and lack of organizational structure at the HCA. The dissemination of national cancer information is performed by the HCA, one of the sources of the Cancer-NIS and not by the Colombian Observatory on Cancer, agency responsible for knowledge management (Fig. 1).

To plan and project strategies and activities for cancer control in the country, it is essential to have valid, comparable, timely and comprehensive information; otherwise it will be impossible to determine the impact of the Ten-year Plan for Cancer Control. This strategic plan aims to reduce mortality in this group of diseases, and it focuses its actions in controlling colon, breast, cervix and prostate cancer; and pediatric acute leukemia. The model that guides this plan aims to control risk, early detection, treatment, rehabilitation and palliative care. The scenarios for the development of actions are at the political, community and health services levels.

Knowledge management is the core of the model through monitoring, research and analysis of the health situation regarding cancer. The Colombian Observatory on Cancer (COC) is the Cancer-NIS entity responsible for making policy recommendations and to provide guidance to the health system based on the information provided by the HCA's cancer registry, the Integral System of Information of Social Protection (SISPRO), and the population-based cancer registries (Fig. 1).

The PBCRs of Cali, Pasto, Bucaramanga and Manizales belong to the International Association of Cancer Registries, and their quality has been endorsed by the IARC. All of them have motivated staff, able to work in coordination with the COC to build the population indicators of incidence, mortality and survival in their coverage areas; figures to take actions is the role of PBCRs in cancer control. This information is essential for tracking, monitoring and evaluating the Ten-year Plan in these municipalities. The PBCRs can be valuable tools for assessing the reliability of the reporting system of cancer in their coverage areas. In addition, they provide inputs to Colombian-NCI , to obtain estimates of the incidence, based on mortality information in most of the Colombian regions that lack cancer registries. With the development and maturation of Cancer-NIS, valuable information will be obtained about the behavior of cancer in Colombia. Meanwhile, the World Health Organization through the IARC will continue to monitor the quality of the Cancer Registries of Colombia, and in turn, these can serve the Ministry of Health to assess the reliability of the reporting cancer system.

 Unfortunately, the Colombian PBCRs are excluded from the current legislation of the MHSS, and do not have a budget allocation. Currently, funding for PBCRs comes from the Universities: (Valle, Nariño, Caldas and, Autonoma de Bucaramanga); with partial support from Colombian-NCI; and some resources of the corresponding local Health Municipalities. It is not feasible or desirable to create a National PBCR for Colombia, but it is a priority to ensure the sustainability of the existing regional PBCRs and to create a few more regional ones. The strategic decision of where to implement and sustain new population-based registries is a pending task of the Colombian health authorities, which should involve epidemiologists and policy makers, to ensure good quality information with sustainability and representativeness of the cancer registries. The PBCRs should be included in the Oncology policy to use efficiently the high quality data they provide on the formulation and evaluation of cancer control programs. Disclosure of information nationwide must be the product of an adequate interpretation and integration of the available information, clearly specifying the existing limitations. Investment in generating quality information is cost-effective; it allows prioritizing the needs, and to invest resources for the benefit of the greatest number of people. Actions to control diseases that are not based on reliable information will ultimately be more costly.
